# Automatically transferring supervised targets method for segmenting lung lesion regions with CT imaging

**DOI:** 10.1186/s12859-023-05435-5

**Published:** 2023-09-04

**Authors:** Peng Du, Xiaofeng Niu, Xukun Li, Chiqing Ying, Yukun Zhou, Chang He, Shuangzhi Lv, Xiaoli Liu, Weibo Du, Wei Wu

**Affiliations:** 1 State Key Laboratory for Diagnosis and Treatment of Infectious Diseases, National Clinical Research Center for Infectious Diseases, Collaborative Innovation Center for Diagnosis and Treatment of Infectious Diseases, The First Affiliated Hospital, School of Medicine, Zhejiang University, 79 QingChun Road, Hangzhou, 310003 Zhejiang China; 2 Hangzhou AiSmartIoT Co., Ltd., Hangzhou, Zhejiang China; 3 Artificial Intelligence Lab, Hangzhou AiSmartVision Co., Ltd., Hangzhou, Zhejiang China; 4 Department of Radiology The First Affiliated Hospital, School of Medicine, Zhejiang University, Hangzhou, Zhejiang China

**Keywords:** Pseudolabel, Dual-branch model, Pulmonary disease, Cost-effective

## Abstract

**Background:**

To present an approach that autonomously identifies and selects a self-selective optimal target for the purpose of enhancing learning efficiency to segment infected regions of the lung from chest computed tomography images. We designed a semi-supervised dual-branch framework for training, where the training set consisted of limited expert-annotated data and a large amount of coarsely annotated data that was automatically segmented based on Hu values, which were used to train both strong and weak branches. In addition, we employed the Lovasz scoring method to automatically switch the supervision target in the weak branch and select the optimal target as the supervision object for training. This method can use noisy labels for rapid localization during the early stages of training, and gradually use more accurate targets for supervised training as the training progresses. This approach can utilize a large number of samples that do not require manual annotation, and with the iterations of training, the supervised targets containing noise become closer and closer to the fine-annotated data, which significantly improves the accuracy of the final model.

**Results:**

The proposed dual-branch deep learning network based on semi-supervision together with cost-effective samples achieved 83.56 ± 12.10 and 82.67 ± 8.04 on our internal and external test benchmarks measured by the mean Dice similarity coefficient (DSC). Through experimental comparison, the DSC value of the proposed algorithm was improved by 13.54% and 2.02% on the internal benchmark and 13.37% and 2.13% on the external benchmark compared with U-Net without extra sample assistance and the mean-teacher frontier algorithm, respectively.

**Conclusion:**

The cost-effective pseudolabeled samples assisted the training of DL models and achieved much better results compared with traditional DL models with manually labeled samples only. Furthermore, our method also achieved the best performance compared with other up-to-date dual branch structures.

## Introduction

The proportion of infected regions of the lung could be used as visual evidence to assist the clinical physician in determining the severity of pneumonia [1, 2]. Furthermore, the progression of lung disease can also be predicted by continuously monitoring the volume and quality of infected regions [3, 4]. For example, the gas volume, tissue mass and recruitability measured by chest computed tomography (CT) scan analysis are important when setting the mechanical ventilation in acute respiratory distress syndrome (ARDS) [5–7].

Since 2020, many studies [8–10] have demonstrated using deep learning (DL) models to automatically segment infected regions of pneumonia with good accuracy. Fan et al. [8] developed a novel COVID-19 lung infection segmentation deep network (Inf-Net) for automatically identifying infected regions from chest CT scans. They achieved a value of 0.739 measured in the mean Dice similarity coefficient (DSC) for the segmentation of infected regions. Yan et al. [9] also investigated the segmentation of infected regions due to COVID-19, and a feature variation block in the segmentation of infected regions was introduced, which could better differentiate the diseased area from healthy areas in the lung. Furthermore, they used more effective progressive spatial pyramid pooling in the feature extraction stage as well. The optimum DSC values achieved in their studies for intact lung and infected regions were 0.987 and 0.726, respectively. Liu et al. [10] employed a two-stage cross-domain transfer learning framework to segment COVID-19 infection regions. This framework took advantage of attention-aware feature fusion and large reception fields for accurate object segmentation. The final experiment acquired a DSC of 0.668. However, these studies suffered from the tremendous effort required to manually annotating large-scale datasets well and achieved relatively low accuracy measured in DSC.

The U-Net [11] network structure together with its descendant family, such as 3D U-Net [12] and V-Net [13], achieved excellent results in the domain of segmentation. In addition, attention networks and transfer learning concepts have also been utilized. As in biological field, transfer learning had been approved effective in cancer detection and prediction using relatively small datasets [14–16]. Till now, most networks in chest CT images required sufficient high-quality labeled samples for DL models to be trained and verified. Since the infected lung regions could be illustrated as ground-glass opacity or consolidation regions and could adhere together with normal tissues on CT images, it would be costly to separate infected regions from healthy lung parenchyma. Furthermore, a set of CT images usually consists of dozens or hundreds of lung image slices, which makes it a very expensive and time-consuming procedure for a professional radiologist to manually annotate chest CT images. Therefore, it was necessary to train a decent DL model with very limited high-quality labeled samples.

Recently, a weak supervised learning frame structure achieved satisfactory results by utilizing noisy or scribble-labeled samples in the training process of models. For example, Luo et al. [17] designed a semi-supervised network with a strong–weak dual-branch structure in the pixel-level segmentation of images. Their dual branch structure handled strong (high quality) samples and weak (noisy) samples to exploit the joint discrimination of strong and weak annotations and brought significant improvements over the previous methods. Luo et al. [18] and Liu et al. [19] initiated effective scribble-supervised networks in medical image segmentation. They employed a dual-branch network with a mixed pseudolabeling strategy to train DL models with scribble annotations. Yang et al. [20] initiated a noise Divergence-Aware Selective Training (DAST) strategy to identify severely noisy annotations and slightly noisy annotations and then treated them differently to improve the noise tolerance of DL models. Tarvainen et al. [21] proposed the mean-teacher framework to improve temporal ensembling [22] for semi-supervised learning. Mean-teacher employed moving-average to update the weights of the teacher network instead of label predictions. This framework outperformed the traditional DL network with a lower error rate by fewer labels on some open sourced databases, such as Street View House Number (SVHN) [23] and ImageNet 2012 [24]. Yu et al. [25] improved Mean-teacher by a novel uncertainty-aware self-ensembling Mean-teacher UA-MT framework to enable the student model to gradually learn from meaningful and reliable targets by exploiting the uncertainty information in left atrium segmentation for 3D magnetic resonance imaging (MRI). Experiments showed that their method achieved high performance gains by incorporating the unlabeled dataset.

In our study, we explored utilizing the inherited Hounsfield unit (Hu) value of CT images to segment infected regions as pseudolabeled samples. Next, these almost zero-cost samples together with a small number of high-quality manually annotated samples were used to train our DL models. A semi-supervised dual branch framework was designed. Two kinds of samples were trained in strong and weak branches. We also implemented a method of automatically transferring supervised targets and dynamically selecting the optimal targets to supervise the weak branch training. This approach was intended to improve model accuracy, prevent overfitting in the initial stage, and eliminate noisy interference in the final stage. Furthermore, we conduct extensive experiments over our proposed methods to verify the effectiveness and experimental results demonstrate that our proposed method achieves state-of-the-art performance under various ratios of annotation noise for universal lung lesion segmentation.

## Materials and methods

### Study dataset and data preprocessing

A total of 869 transverse-section CT samples were collected from 869 patients with lung infections, such as COVID-19 (539, 62.0%), influenza pneumonia (100, 11.5%) and tuberculosis (230, 26.5%). In total, 850 cases were from the First Affiliated Hospital of Zhejiang University, and 19 cases were from an internet open source of the COVID-19 database [26]. The latter dataset contains 20 labeled COVID-19 CT scans in total. However, the infected region of one case (radiopaedia_29_86490_1.nii.gz) only occupied 0.014% of the entire lung, which was too insignificant for our further verification. Thus, we removed this case from the external verification benchmark. All CT imaging was in the format of digital imaging and communications in medicine (DICOM) with 5 mm thickness between slices.

**Fig. 1:**
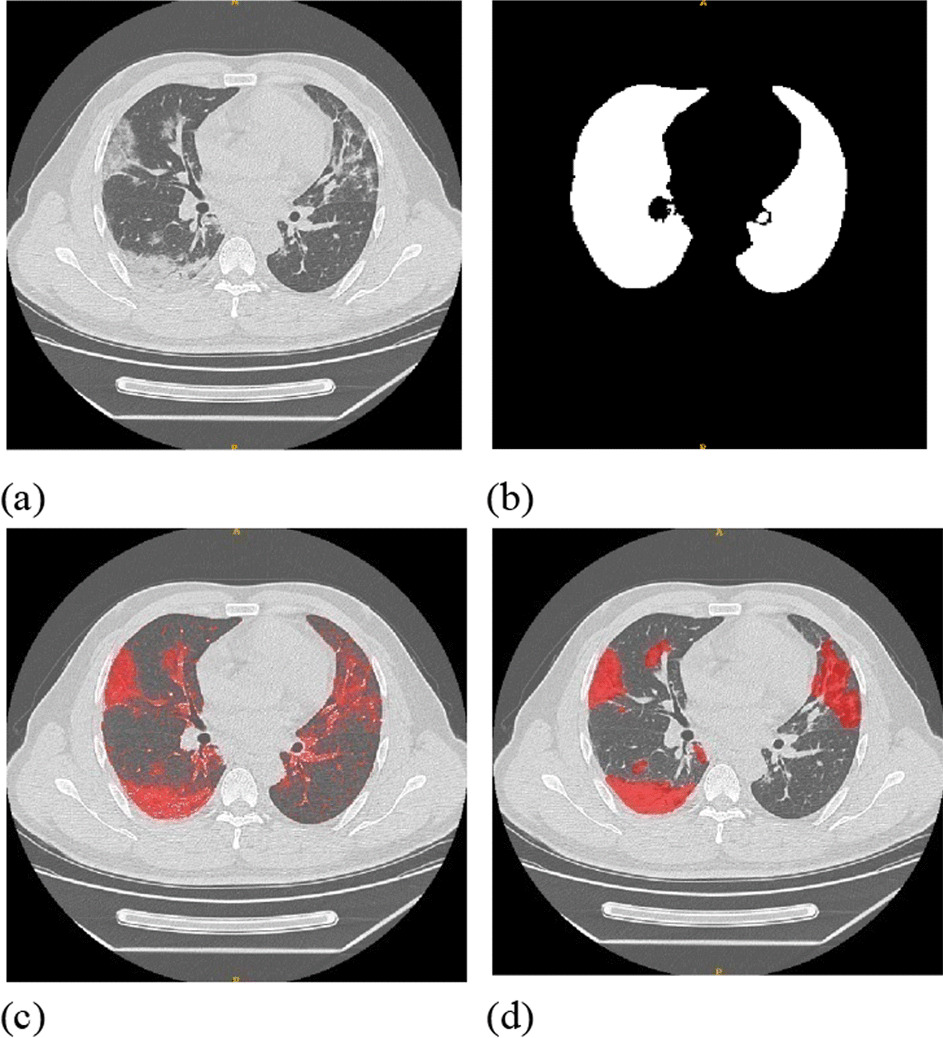
Typical Hu-based pseudo-labeled and manually labeled CT images: **a** original CT image with pneumonia; **b** generated mask of intact lung; **c** pseudo-labeled (Hu_[− 750,50]_); **d** manually annotated by radiologists

In our study, 250 CT datasets were manually annotated to the infected regions (image d in Fig. 1) by two professional radiologists, in which 200 sets were randomly selected to be used in the training process and the remaining 50 were used for the test set. The next 600 CT datasets were automatically segmented based on their Hu values, and these cost-effective samples were used to assist in model training. The last 19 fine annotated CT datasets from open source were utilized as an external test benchmark.

As the digital grayscale image had a pixel value ranging from [0, 255], the raw CT data were converted from Hu to the interval of the aforementioned values accordingly. The Hu data matrix was clipped within [− 1200, 600] (any value beyond this was set to − 1200 or 600 accordingly) and then linearly normalized to [0, 255] to fit into the digital image format for further processing.

### Dataset preprocess to generate the mask for intact lung

The lung CT images were preprocessed to generate the mask for the intact lung (image b in Fig. 1), following the method reported by Hofmanninger et al. [27, 28], which was based on a U-Net DL model. They trained the model with a total of 121,820 CT image slices that were annotated with two radiologists and obtained an accuracy of 0.97 measured in DSC. As this method achieved steady and satisfactory results, the rest of our study focused on the segmentation of infected regions.

### Utilize the value of Hu to generate cost-effective pseudo-labels

To further utilize unlabeled CT image dataset collections, we employed the inherent Hu value to generate noisy samples. These nearly zero-cost "dirty" datasets were used to assist the training of our dual-branch model. The segmentation of noisy infected regions was based on different valves of Hu values. Tang et al. [29] divided different infection regions in the lung with (mild) ground-glass opacity (GGO) and (severe) consolidation, which is defined with the Hu value. This value of the ground-glass opacity region was [− 750, − 300], and consolidation was [− 300, 50]. Thus, for one set of CT images, we generated pseudolabeled samples accordingly as Hu_[_− _750,50]_, which are shown in (c) of Fig. 1. However, there are some healthy tissues for which the Hu value is also located within [− 200, 50]. Thus, the samples segmented by the Hu value would also include these normal regions as noisy.

### DL model training process

#### ATST method network structure

**Fig. 2:**
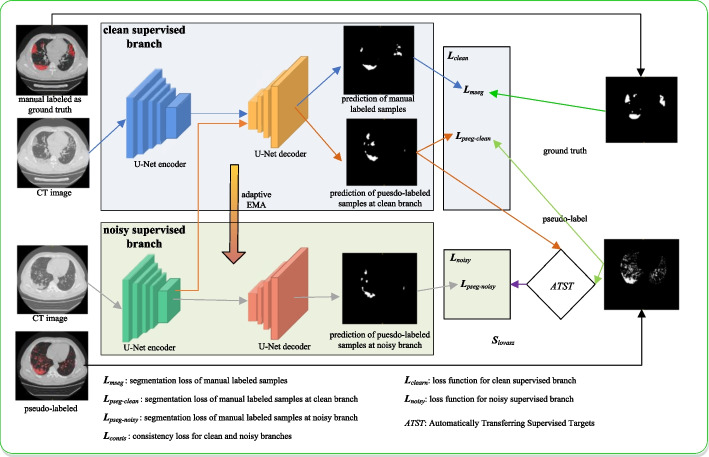
Overview of the proposed cost-effective sample assisted dual-branch framework

Four widely used DL frameworks were explored in our study, including U-Net [11], uncertainty-aware self-ensembling mean teacher (UA-MT) [25], Divergence-Aware Selective Training (DAST) [20] and our automatically transferring supervised targets (ATST) training method, as shown in Fig. 2 U-Net(2015) was the fundamental network in the domain of medical image segmentation, and the UA-MT(2019) and DAST(2022) structures were more up-to-date semi-supervised frameworks. UA-MT enhanced the mean-teach network [21] by using the Monte Carlo sampling method to predict the uncertainty for each label to screen out unreliable samples. DAST further designed a divergence-aware selective training strategy to separate severely and slightly noisy annotations during the training process.

A dual-branch (clean and noisy supervised branches) structure was used, as there were two kinds of samples in our study. Minorities of datasets were carefully annotated by experienced radiologists, and the remaining majorities were zero-cost labels from the automatic segmentation of the Hu value of CT images. The backbone encode-decode structure of both branches was based on U-Net, which included two network paths: contracting and expanding. The images were first fed into the contracting path to finish the down sampling or encoding process and capture the context information. Then, the up sampling or decoding process was completed in the symmetrical expanding path to obtain precise localization information of the targets.

Furthermore, the theory of the mean teacher semi-supervised training process was employed to utilize noisy samples as supplementary datasets to improve the major (clean supervised) branch. Inherited from the mean-teacher methodology, the clean supervised model learned from the noisy supervised model by minimizing the segmentation loss on the labeled data and the consistency loss with respect to the targets from the noisy supervised model on all input data. In addition to the supervised reverse gradient update process, we enhanced the idea of the exponential moving average (EMA) in Mean-teacher [12] by incorporating adaptive weight updating methods. The traditional EMA function was as follows:

1 $$\theta_{t}{\prime} = \varepsilon \times \theta_{t - 1}{\prime} + (1 - \varepsilon ) \times \theta_{t}$$

where *θ* and *θ'* are the weights of the clean and noisy supervised models, respectively. The noisy supervised weights *θ't* were updated at training step *t*. The smoothing coefficient parameter *ε* was used to control the updating rate. According to Tarvainen's previous work [12], the performance was the best with *ε*= 0.99 in the ramp-up stage and *ε*= 0.999 for the rest.

Our adaptive EMA gradually updates the coefficient *ε* during training based on the following function:

2 $$\varepsilon = 1 - 0.2 \times e^{{\frac{ - 8i}{{iters}}}} (i \in (0,iters))$$

where *iters* is the number of maximum iterations.

The coefficient *ε,* which was initially equivalent to 0.8, allowed more space for the updating of weights to learn from the clean supervised branch quickly and then increased gradually to approach 1 infinitely in the final stage to restrain the turbulence from "dirty" samples.

The ATST module stands for Automatically Transferring Supervised Targets in the early stages of training, the weakly supervised branch mainly relies on labels based on Hu values for supervised learning. However, as training progresses, the strongly annotated branch has gained a certain level of accuracy. At this point, the labels based on Hu values not only do not help the network's learning, but the noise they contain has had a negative impact on the network structure, To fully utilize the unannotated data for training, this paper proposes a Lovasz-based scoring method to evaluate whether the model's predictions are better than those based on Hu values. When the Lovasz score exceeds a certain threshold, the label values of the weakly supervised branch are switched to the noisy data passed through the output values of the strongly supervised branch. At this point, it is believed that the model's predictions of the noisy data are already superior to the labels based on Hu values. Therefore, the weakly supervised branch can use this data for further learning. As the model's accuracy improves, the guidance provided by the weakly labeled data becomes more accurate, which enables the network to fully explore the value of unannotated data. Therefore, the output of the ATST module is the weakly supervised target ground truth (GT) result, as shown in the following equation:

3 $$GT = \left\{ \begin{gathered} P_{pseg - clean} ,S_{lovasz} - \tau \ge 0 \hfill \\ GT_{pseudo - label} ,S_{lovasz} - \tau < 0 \hfill \\ \end{gathered} \right.$$

we define τ as the h-th percentiles of Slovasz values during a certain number (e.g., 100) of iterations, respectively, The *S*_*lovasz*_ calculation formula is:

4 $$S_{lovasz} { = }\frac{{LEV(GT_{pseudo - label} ,P_{pseg - clean} )}}{{e^{{div(GT_{pseudo - label} ,P_{pseg - noisy} )}} }}$$

*LEV* is lovasz-extension value [30], *div*(·) represents a symmetric divergence function between two predictions.

#### Loss functions

The clean branch network was optimized by minimizing the loss function *L*_*clean*_, which consisted of the manually labeled image segmentation loss *L*_*mseg*_, the pseudolabeled image segmentation loss in clean branch *L*_*pseg-clean*_, which was calculated as follows:

5 $$L_{clean} = L_{mseg} + \lambda \times L_{pseg - clean}$$

The loss function for the noisy branch network was the pseudo-labeled image segmentation loss *L*_*pseg-noisy*_, which was:

6 $$L_{noisy} = \lambda \times L_{pseg - noisy}$$

*L*_*mseg*_, *L*_*pseg-clean*_ and *L*_*pseg-noisy*_ were standard segmentation loss *L*_*seg*_ including dice loss *L*_*dice*_ and binary cross-entropy loss *L*_*BCE*_.

7 $$(L_{mseg} ,L_{pseg - clean} ,L_{pseg - noisy} ) \in L_{seg} = 0.5 \times (L_{dice} + L_{BCE} )$$

### Algorithm details

The procedure of the proposed ATST method network is listed in Algorithm 1.
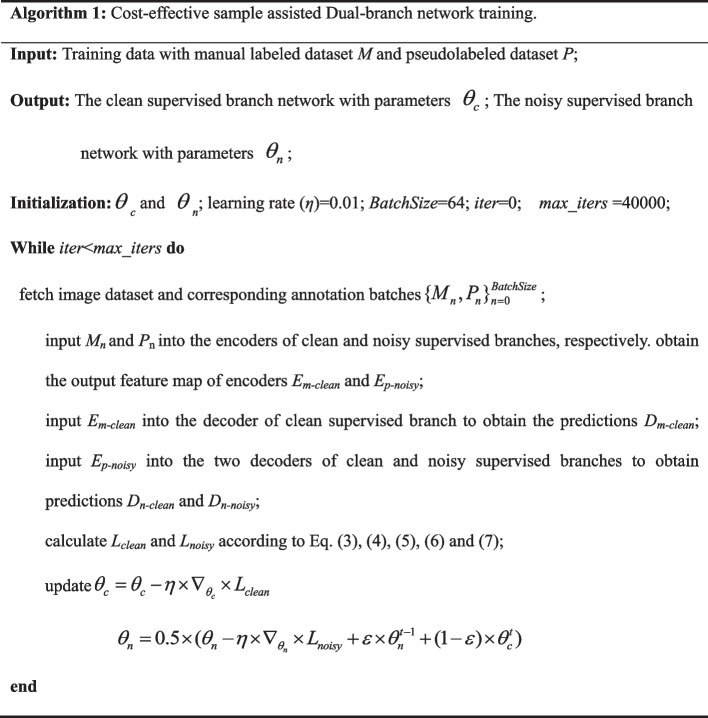


## Experiment and results

### Implementation

An Intel i7-8700k CPU together with an NVIDIA RTX3090 24 GB GPU card was used as the training server. Our approach was implemented with PyTorch and iterated with the stochastic gradient descent (SGD) optimizer 40K times. The initial learning rate was set to 0.01 and decayed exponentially. All input images were normalized to 256 × 256 (pixels), and the batch size was set to 128, which included 64 manually annotated samples and 64 pseudolabeled samples. At the same time, generic data augmentation mechanisms, random clipping and left–right flipping were performed on specimens to increase the number of training samples and prevent data overfitting.

### Evaluation criteria

Different measurements were used to evaluate the performance of the proposed methods, including the dice similarity coefficient (DSC) and Hausdorff distance (HD). DSC is the most widely used criterion, which measures the similarity between the ground truth and the prediction score maps and is calculated as follows:

8 $$DSC(A,B) = \frac{{2|{\text{A}} \cap {\text{B}}|}}{{|{\text{A}}| + |{\text{B}}|}} \times 100\%$$

where A is the contour of the segmented region and B denotes the ground truth. The DSC is a value between [0, 1]. The larger the value, the better the segmentation effect achieved.

DSC is sensitive to the internal filling of the mask, while HD is more sensitive to the segmented boundary. HD is a distance measurement between the contour of the segmentation result and the contour of the ground truth label. Usually, HD95 is used instead, which is the 95th percentile of the maximum HD to eliminate the impact of a very small subset of the outliers. The unit of HD is pixel. The smaller the value is, the closer the segmentation result is to the ground truth and the better the segmentation effect, which is calculated as follows:

9 $$H(A,B) = \max (h(A,B),h(B,A))$$

where

10 $$h(A,B) = \mathop {\max }\limits_{a \in A} \left\{ {\mathop {\min }\limits_{b \in B} \left\| {a - \left. b \right\|} \right.} \right\}$$

11 $$h(B,A) = \mathop {\max }\limits_{b \in B} \left\{ {\mathop {\min }\limits_{a \in A} \left\| {b - \left. a \right\|} \right.} \right\}$$

### Ablation study

We conducted ablation experiments using all the manually labeled data and 400 pseudo-labeled data. We further investigated the effect of using different methods for the dual-branch network: (1) using a traditional single-branch model (U-Net) and training only with manually labeled data; (2) Adaptive EMA, which encourages automatically adjusting the update weight of strong and weak branches; (3) using our ATST method for dual-branch strong–weak supervision training. The quantitative evaluation results were presented in Table 1. It can be observed that training the model using only manually labeled data with a single-branch network resulted in a lower accuracy. Additionally, the effect of using Adaptive EMA was better than without EMA. The results indicated that our proposed ATST dual-branch network fully utilized the pseudo-labeled data and achieved optimal results.

**Table 1:** Ablation study of our automatically transferring supervised targets methods, where " + " and "**−**" meant with and without ATST operation

Method (manual labeled: pseudo labeled)	Tested with 50 benchmark		Tested with external 19 benchmark	
	DSC (%)	95HD (voxel)	DSC (%)	95HD (voxel)
U-Net (200:0)	70.02 ± 20.59	29.97 ± 46.38	69.30 ± 12.29	25.29 ± 25.31
EMA (200:400)	76.19 ± 27.12	27.29 ± 31.18	75.89 ± 18.17	25.04 ± 25.34
Adaptive EMA (200:400)	78.34 ± 22.34	25.02 ± 30.92	76.29 ± 16.74	21.44 ± 24.89
−ATST (200:400)	80.87 ± 13.24	13.71 ± 18.28	80.91 ± 11.26	15.72 ± 15.98
+ ATST(ours) (200:400)	**83.56 ± 12.10**	**11.19 ± 16.41**	**82.67 ± 8.04**	**12.69 ± 11.14**

Bold font indicated the best results obtained

### Segmentation of infected regions

Our proposed model was compared with 3 other state-of-the-art DL models: U-Net, UA-MT and the recently developed DAST. All four models were trained with a fixed number of manually annotated sample base cohorts together with pseudolabeled samples. For the U-Net model, we added the choice of training with the 200 base cohorts only. In contrast, the remaining 3 DL models all had dual branch structures. The models were naturally fed by two different kinds of training datasets and could not be trained with the base cohort only. We investigated the performance of these four methods under different pseudolabeled sample ratios: (1:0.5); (1:1); (1:2) and (1:3) together with the base cohort. Table 2 shows the comparison results on our internal (50 cases) set as well as the publicly available external (19 cases) benchmarks, measured in DSC and 95HD.

**Table 2:** Comparison results of the segmentation of infected regions on our internal (50 cases) and external (19 cases) benchmarks

Method	#Pseudolabel	Tested with 50 benchmark		Tested with external 19 benchmark	
		DSC (%)	95HD (voxel)	DSC (%)	95HD (voxel)
U-Net	**0**	**70.02 ± 20.59**	**29.97 ± 46.38**	**69.30 ± 12.29**	**25.29 ± 25.31**
	100 (1:0.5)	68.29 ± 23.23	30.29 ± 45.65	68.22 ± 13.82	25.98 ± 27.72
	200 (1:1)	67.87 ± 23.88	31.14 ± 45.12	63.51 ± 12.01	26.85 ± 28.10
	400 (1:2)	64.16 ± 25.12	33.64 ± 37.32	60.23 ± 15.62	28.33 ± 29.52
	600 (1:3)	59.47 ± 26.89	34.55 ± 45.65	58.40 ± 15.47	30.49 ± 28.73
UA-MT	100 (1:0.5)	76.71 ± 22.78	30.32 ± 35.54	73.69 ± 14.51	16.43 ± 18.89
	200 (1:1)	77.02 ± 19.99	29.12 ± 34.28	75.15 ± 11.34	15.67 ± 16.74
	**400 (1:2)**	**78.36 ± 20.31**	**27.76 ± 36.57**	**76.10 ± 10.09**	**13.40 ± 14.23**
	600 (1:3)	78.22 ± 19.69	28.31 ± 35.92	76.27 ± 10.21	12.87 ± 13.17
DAST	100 (1:0.5)	79.28 ± 15.72	17.23 ± 18.19	76.45 ± 12.36	14.35 ± 16.81
	200 (1:1)	80.19 ± 13.96	15.82 ± 16.29	78.60 ± 11.62	13.21 ± 15.54
	400 (1:2)	80.41 ± 14.20	15.60 ± 17.83	79.72 ± 11.73	15.43 ± 13.39
	**600 (1:3)**	**81.54 ± 13.58**	**14.68 ± 15.36**	**80.54 ± 10.54**	**16.69 ± 14.28**
ATST	100 (1:0.5)	79.34 ± 13.58	15.81 ± 30.78	79.45 ± 10.07	11.35 ± 10.92
	200 (1:1)	82.46 ± 14.07	14.41 ± 26.18	80.60 ± 9.33	13.21 ± 9.14
	**400 (1:2)**	**83.56 ± 12.10**	**11.19 ± 16.41**	**82.67 ± 8.04**	**12.69 ± 11.14**
	600 (1:3)	82.82 ± 13.38	12.85 ± 20.29	81.72 ± 9.29	13.43 ± 11.72

The results are expressed as the mean ± standard deviation. The default # of manual annotated samples is 200. Bold font indicated the best results obtained for each algorithm

**Fig. 3:**
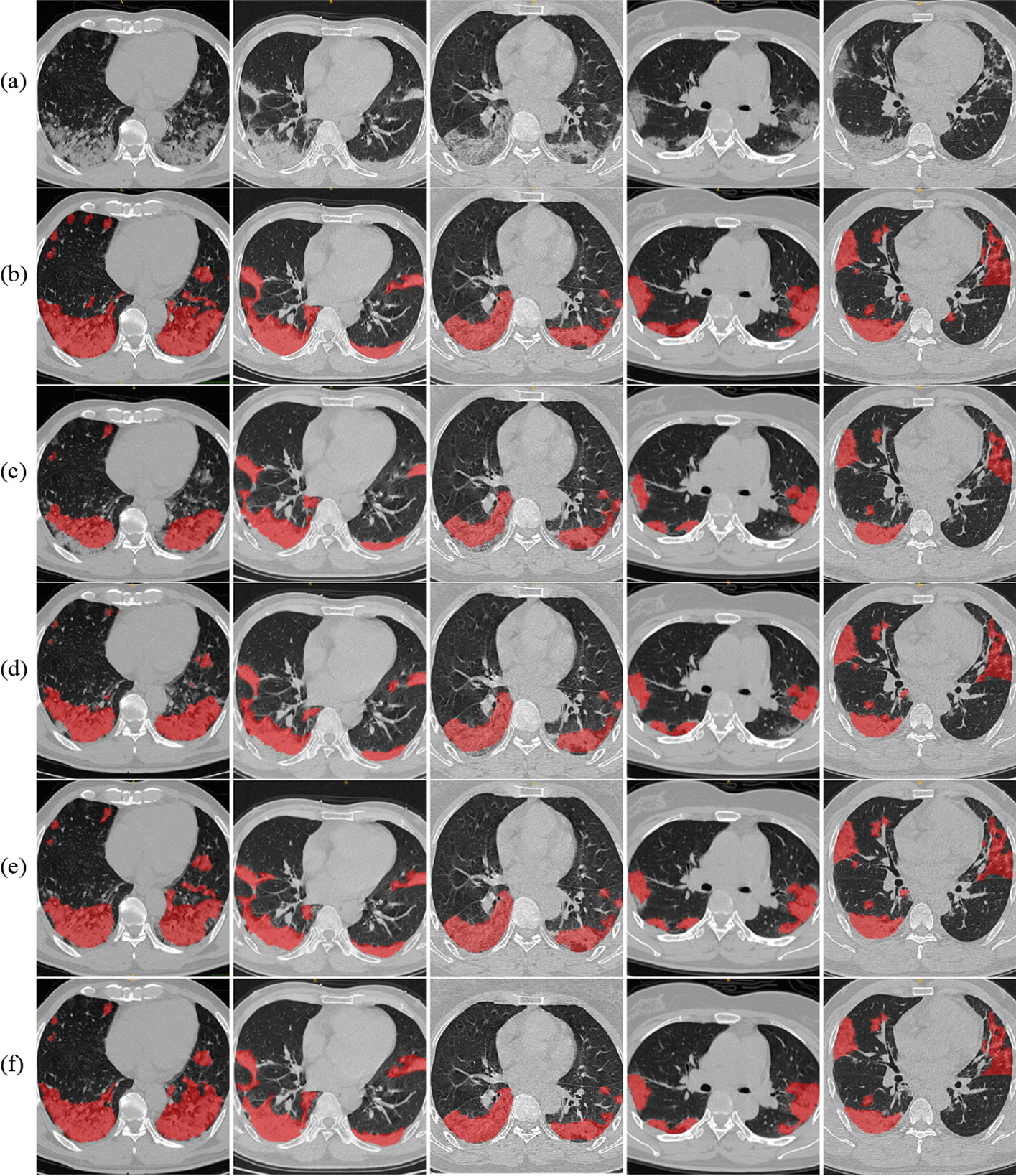
Three scan slices were selected to demonstrate the difference of each competing method. **a** original CT; **b** ground truth (manually annotated); **c** U-Net labeled; **d** UA-MT labeled; **e** DAST labeled; **f** ATST labeled. Column 1, 2 and 3 were samples from internal dataset. Column 4 and 5 were samples from external dataset

The most fundamental method was trained by U-Net. It can be observed that the leading results were achieved with the 200 base cohorts only, and the more pseudolabeled samples added (randomly mixed), the more unsatisfied results were obtained. Simply adding relatively inaccurate samples to the training set would not lead to a satisfactory outcome. For the remaining dual branch models, pseudolabeled samples greatly assisted the original training cohort in reaching superior results compared with the U-Net baseline. It was observed that UA-MT and ATST achieved the best performance with a ratio of 1:2, and for DAST, it was 1:3. We believed the DAST had a selective mechanism to discard some poor training samples. Among the competitors, our proposed method achieved the best performance. The final results reflected 2.02% improvement on 50 benchmarks and 2.13% on 19 benchmarks measured in DSC compared with most up-to-date algorithms. Figure 3 provides a visualization of the segmentation results for the comparison methods. It was demonstrated that our proposed method ATST can achieve results that were closer to the manually labeled ground truth results than other algorithms.

Table 3 analyzed the number of parameters for each model and the time required for training and testing. During forward prediction, since each CT set contains a different number of slices, we calculated the time required for different models to predict a single slice in order to compare their time. According to the results, the parameter count of the dual-branch network structure is generally higher than that of the single-branch network, so its training time is longer. However, when we perform forward prediction on the dual-branch network, we only use the parameters of the strong branch. Under the same basic network, the time consumption is similar.

**Table 3:** Comparative results on parameters and time-consumption with other state-of-the-art methods.

Method	# of parameters	Overall training time (minutes)	Time-consumption per slice (ms)
U-Net	1.8 × 10^6^	506	81
UAMT	3.6 × 10^6^	877	82
DAST	9.1 × 10^6^	1280	95
ATST (ours)	3.6 × 10^6^	1365	82

### Qualitative and quantitative analysis of infected regions

**Fig. 4:**
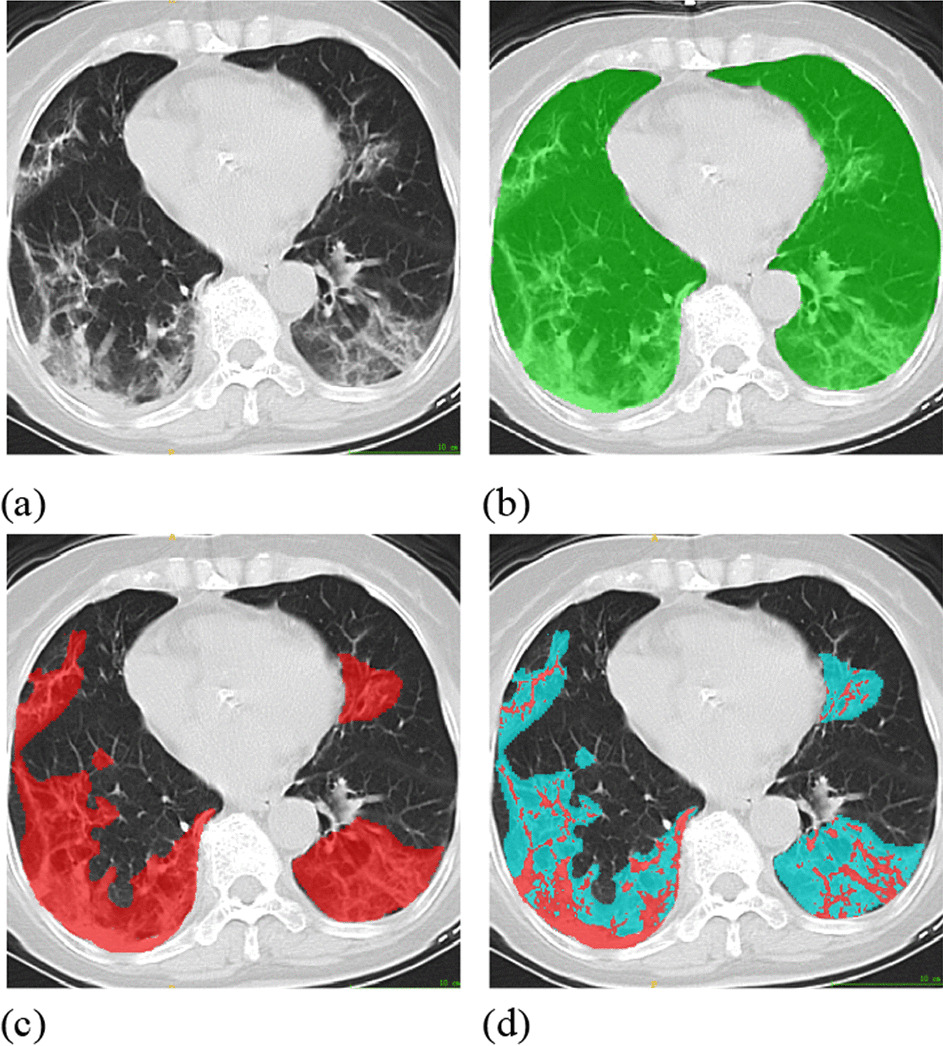
**a** Original lung CT image; **b** effective region (or mask of lung); **c** infected region; **d** infected region classified by GGO (blue, Hu_[− 750,300]_) and consolidation (light red Hu_[− 300,50]_)

After automatic segmentation of the mask of the lung and the infected regions for each slice of the lung CT image, the model could sequentially calculate the volume of the lung and the infected regions, as well as the ratio of the infected regions in the total lung. Furthermore, quantitative analysis of the infected regions could be concluded accordingly. For example, the two main features of COVID-19, groundglassopacity(Hu_[− 750,300]_)andconsolidationlesions(Hu_[− 300,50]_), could be visualized for evaluation inside the infection region by our model, as shown in Fig. 4.The qualitative and quantitative analysis of infected regions may help physicians with the diagnosis, prognosis and follow-up of patients.

Discussion & conclusions

With the rapid development of artificial intelligence technology, the expertise of professional radiologists, such as the segmentation of medical images, could be injected into deep learning models to generate a quantitative analysis report automatically [31, 32]. So far, the most challenging work in DL model training is the annotation of a large amount of fine-quality pixel-level images [4]. As for chest CT images, how to present an approach that autonomously identifies and selects the optimal target of infected regions for the purpose of enhancing learning remains a clinical problem.

In this study, we utilized the intrinsic Hu value of CT images to create pseudo-labels, which assisted the training of our segmentation models. Even though they were "dirty" samples, these cost-effective datasets greatly improved the result of the state-of-the-art segmentation algorithm by 13.54% from U-Net, which uses manually annotated samples only, as measured by DSC. Our method also achieved the best performance compared with other up-to-date dual branch structures.

However, this study had several limitations. In some cases, the segmentation models would identify healthy tissues together with valid infected regions. In addition, some ground-glass opacity infected regions that were barely noticed by human eyes seemed too tenuous to be captured by the segmentation model in this study. Therefore, the corresponding mask in such a scenario deviates from the ground truth. In addition, more pneumonia cases from different subtypes should be included to promote the accuracy of segmentation. For example, some atypical infection signs, such as pleural effusions, cannot be distinguished with our methods.

In conclusion, this study facilitates the qualitative and quantitative analysis of infected regions of the lung, which could be used as visual evidence to assist clinical physicians. In the future, doctors may carry out a quantitative analysis of the severity of pneumonia patients with this model only or combined with other clinical data, such as the blood oxygenation index. At the same time, they can compare the sequential CT scans of the same patient to estimate the progression of disease and provide reliable evidence for further treatment.

## Data Availability

All data included in this study are available from the corresponding author upon reasonable request.
